# Simulation-based prior knowledge elicitation for parametric Bayesian models

**DOI:** 10.1038/s41598-024-68090-7

**Published:** 2024-07-27

**Authors:** Florence Bockting, Stefan T. Radev, Paul-Christian Bürkner

**Affiliations:** 1 Department of Statistics, TU Dortmund University, Dortmund, Germany; 2 Cognitive Science Department, Rensselaer Polytechnic Institute, Troy, NY USA

**Keywords:** Psychology, Statistics, Computational science

## Abstract

A central characteristic of Bayesian statistics is the ability to consistently incorporate prior knowledge into various modeling processes. In this paper, we focus on translating domain expert knowledge into corresponding prior distributions over model parameters, a process known as prior elicitation. Expert knowledge can manifest itself in diverse formats, including information about raw data, summary statistics, or model parameters. A major challenge for existing elicitation methods is how to effectively utilize all of these different formats in order to formulate prior distributions that align with the expert’s expectations, regardless of the model structure. To address these challenges, we develop a simulation-based elicitation method that can learn the hyperparameters of potentially any parametric prior distribution from a wide spectrum of expert knowledge using stochastic gradient descent. We validate the effectiveness and robustness of our elicitation method in four representative simulation studies covering linear models, generalized linear models, and hierarchical models. Our results support the claim that our method is largely independent of the underlying model structure and adaptable to various elicitation techniques, including quantile-based, moment-based, and histogram-based methods.

## Introduction

The essence of Bayesian statistics lies in the ability to consistently incorporate prior knowledge into the modeling process^1,2^. The specification of sensible prior distributions over the parameters of Bayesian models can have multiple advantages including improved convergence, sampling efficiency, parameter recoverability, and predictive performance^3–6^.

Despite these apparent advantages, it is often unclear a priori what constitutes a “sensible” prior^7^. In this paper, we focus on the elicitation and translation of expert knowledge into prior distributions, also known as *prior elicitation*^5^. Against this background, *a sensible prior is one that accurately reflects domain knowledge as elicited from an expert or a group of experts*. However, meeting this criterion presents its own set of challenges: Model parameters for which priors are needed might lack intuitive meaning for the domain expert^8^ and the relationship between priors and the data may not be apparent from the model, especially for complex models^9^. Moreover, constructing priors for every single model parameter in models with a large number of parameters might be inefficient or even infeasible.

To address these challenges, several tools for prior elicitation have been developed in the past^5,6,10–16^. Despite the widespread application of Bayesian statistics nowadays, the field of prior elicitation still lags behind in terms of its routine implementation by practitioners. One contributing factor is that many existing methods primarily aim to elicit information about the model parameters directly. This approach makes these methods inherently model-specific, limits their widespread applicability, and poses a challenge for experts in terms of interpretability^9,17,18^.

In recent years, there has been an increasing focus on the development of model-agnostic approaches that center around the prior predictive distribution^6^. These methods allow for the integration of expert knowledge regarding observed data patterns (i.e., elicitation in the observable space). In contrast to interpreting model parameters, domain experts can usually effectively interpret the scale and magnitude of observable quantities^5,9,16,19,20^. Despite these recent developments, the general applicability as well as the actual application of elicitation methods remain limited^5^. This lack of popularity persists, at least in part, because existing methods are still relatively complex, do not easily generalize to different types of expert information, or necessitate substantial tuning or other manual adjustments. In light of the preceding considerations, we introduce an elicitation method that seeks to overcome these challenges. Specifically, this work makes a contribution to prior elicitation research by proposing a method that satisfies the following criteria:

1. *Model independence* Our method is agnostic to the specific probabilistic model, as long as sampling from it is feasible and stochastic gradients can be computed.
2. *Effective utilization of expert knowledge* By incorporating diverse expert information on model parameters, observed data patterns, or other relevant statistics, our method maximizes the utility of expert knowledge.
3. *Flexibility in elicitation techniques* Our method can adapt to different elicitation techniques, ensuring that individual expert preferences are considered.
4. *Modular design* Due to its modular structure our method allows easy adaptation, improvement, or replacement of specific components, both during method development and application.

## Related work

The process of prior elicitation involves the extraction of expert knowledge and its translation into corresponding prior distributions for the parameters in probabilistic models^5,10,11,14^. Knowledge extraction can incorporate asking an expert directly about the probability distribution of the model parameters or indirectly about other quantities that may be easier for the expert to understand^11,21,22^. These quantities include observable data patterns (i.e., variables in the data space such as expected mean responses) as well as familiar statistics derived from the predictive distribution of the outcome variable (e.g., the percentage of variance explained).

As interpretability is an essential requirement for elicited quantities, it has been argued that asking about parameters is only meaningful if they can be interpreted in terms of a limiting average of observables^5,23^. That said, experts may also have knowledge about parameter values through prior studies, meta-analyses, and similar sources, which do not necessarily require easy interpretability. A thorough discussion of the interpretability of various elicited quantities is beyond the scope of this paper but is discussed in detail elsewhere^11,14,21,22^.

Based on their comprehensive review, Mikkola et al.^5^ recently advocate that an elicitation method should include both a model’s parameter and observable space, exhibit model-agnostic characteristics, and prioritize sample efficiency to minimize the human effort involved. Taking these desiderata into consideration, our method builds upon recent advancements in prior elicitation, specifically on the works of Hartmann et al.^16^, da Silva et al.^9^, and Manderson & Goudie^6^. All three methods are model-agnostic approaches that focus (mainly) on eliciting expert knowledge in the observable space but differ in their specification of target quantities, discrepancy measures, and the specific optimization procedure.

Manderson & Goudie^6^ use multi-objective Bayesian optimization, while our approach employs stochastic gradient-based optimization in line with the methods proposed by da Silva et al.^9^ and Hartmann et al.^16^. All three methods, including ours, support quantile-based elicitation. However, our method goes a step further by also allowing histogram or moment-based elicitation. While all of the considered methods allow for eliciting expert information about observable variables, da Silva et al.^9^ additionally supports querying experts with respect to the parameter space. Our method follows this approach and enables the elicitation of expert knowledge about model parameters, observable quantities, and quantities derived from observable quantities (e.g. percentage of variance explained). As such, our method allows for the elicitation of model parameters and observable quantities, both directly and indirectly, thus extending beyond elicitation in the parameter and observable space. Finally, an essential feature of our method is the use of simulations to obtain prior hyperparameter inference, which classifies it as a variant of *simulation-based inference* (SBI)^24^.

## Simulation studies

In this section, we present four simulation studies demonstrating the performance of our elicitation method. We showcase our method using a normal linear regression model in simulation study 1 (Section "Simulation study 1: normal linear regression"), a binomial regression with logit link in simulation study 2 (Section "Simulation study 2: GLMs—binomial model" ), a Poisson regression with log link in simulation study 3 (Section "Simulation study 3: GLMs—Poisson model"), and a multilevel model with normal likelihood in simulation study 4 (Section "Simulation study 4: hierarchical model"). All code and material can be found on GitHub https://github.com/florence-bockting/PriorLearning, our project website https://florence-bockting.github.io/PriorLearning/index.html, and our simulation results in https://osf.io/rxgv2.

### General setup

*Learning algorithm* In each simulation study, we utilize mini-batch stochastic gradient descent to learn all model hyperparameters. Each optimization process is characterized by a set of *algorithm parameters* including the batch size (*B*) , the number of epochs (*E*) , the number of samples from the prior distributions (*S*) , and the initial learning rate ($\phi ^0$) of the cosine decay schedule with restarts used with the Adam optimizer. The specific settings of the optimization process are fully described in the respective sections. All simulation studies were implemented in Python, utilizing the *TensorFlow* library^25^, and optimization was executed on the Linux HPC cluster at Technical University Dortmund (LiDO3) on high-end GPUs (NVIDIA Tesla K40). We note that our methods can also be easily run on the CPUs of common consumer laptops, where they rarely required more than 30 to 60 minutes (often less) until convergence; at least for the models investigated in our simulation studies. More details for each simulation study (e.g. computing time) can be found in the appendix.

*Method verification* An essential property of any method is its validity. In the following simulation studies, we aim to demonstrate the validity of our proposed method, which we define as the method’s ability to recover a hypothetical ground truth. To achieve this, we use the following approach: First, we define a unique hyperparameter vector $\lambda ^*$ that represents the hypothetical ground truth. Conditional on $\lambda ^*$, observations are simulated from the generative model, and predefined target quantities, along with the corresponding elicitation techniques, are computed. The resulting elicited statistics encode the ground truth. Consequently, a *valid* method should be able to learn $\lambda ^*$ when trained on these elicited statistics.

However, this approach has a caveat: learning unique prior distributions from elicited statistics becomes increasingly challenging as model complexity grows^21^. Since the outcome variable generally has lower dimensionality than the model parameters for which we aim to learn prior distributions^6^, a specific set of elicited statistics may correspond to many equally valid priors and thus varying $\lambda '$. This makes it difficult to determine whether the method can recover $\lambda ^*$. As a consequence, for each simulation study, we constructed a set of elicited statistics that, on the one hand, conveys sufficient information to approximately ensure model identification and, on the other hand, is as small as possible.

*Selection of target quantities and elicited statistics* To demonstrate the flexibility of our method in selecting target quantities and elicitation techniques, we utilized the following target quantities in the subsequent simulation studies: model parameters, prior predictions of the outcome variable, and statistics derived from these prior predictions (e.g., $R^2$). Regarding elicitation techniques, we employed quantile-based, histogram-based, and moment-based elicitation. For quantile-based elicitation, the quartiles $Q_p$ with $p=(0.25, 0.5, 0.75)$; for moment-based elicitation, the mean and standard deviation of the target quantity; and for histogram-based elicitation, a histogram comprising *S* observations were used. Further specifications are provided in each simulation study.

### Simulation study 1: Normal linear regression

*Setup* The first simulation study is presented along with an example inspired by a study from Unkelbach & Rom^26^. In this study, participants encounter general knowledge statements in two consecutive phases, during the second of which they must indicate whether each statement is true or false. The main objective is to investigate the influence of two factors on the proportion of true judgments (PTJs): (1) repetition (ReP), which involves presenting some statements from the first phase again in the second phase, and (2) encoding depth (EnC), whereby participants are randomly assigned to groups that differ in the level of elaboration required when processing the statements during the first phase. We consider a 2 (ReP: *repeated, new*) $\times$ 3 (EnC: *shallow, standard, deep*) between-subject factorial design with treatment contrasts for both factors. The baseline levels are *new* for ReP and *deep* for EnC. Following Unkelbach & Rom^26^, we use a linear regression model to describe the data-generating process

1 $$\begin{aligned} y_i&\sim \text {Normal}(\theta _i, s) \\ \theta _i&= \beta _0 + \beta _1x_1 + \beta _2x_2 + \beta _3x_3 + \beta _4x_4 + \beta _5x_5\\ \beta _k&\sim \text {Normal}(\mu _k, \sigma _k) \quad \text { for }k=0,\ldots ,5\\ s&\sim \text {Gamma}(\alpha , \beta ).\\ \end{aligned}$$

The responses $y_i$ for each observation $i=1, \ldots , N$ are normally distributed with mean $\theta _i$ and standard deviation *s*. The expected value $\theta _i$ is modeled as a linear function of ReP and EnC. The regression coefficients $\beta _k$ for $k=0,\ldots ,5$ are assigned normal prior distributions. The standard deviation *s* of the normal likelihood follows a Gamma prior with concentration parameter $\alpha$ and rate parameter $\beta$. The goal is to learn a total of 14 hyperparameters, $\lambda = (\mu _k, \sigma _k, \alpha , \beta )$.

**Figure 1:**
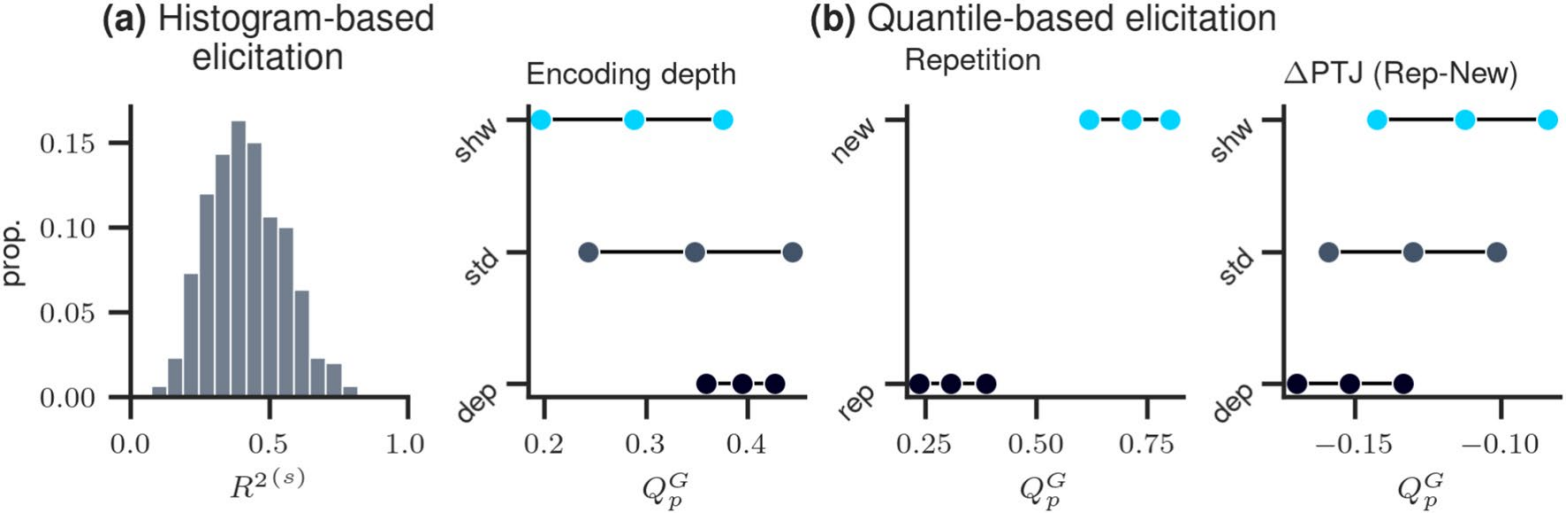
Elicited statistics conditional on $\lambda ^*$. (**a**) elicited histogram of $R^2$; (**b**) three elicited quantiles for each remaining target quantity (see text for detailed information). Abbreviations: For the factor *Encoding depth*: dep-deep, std-standard, and shw-shallow and for the factor *Repetition*: rep-repeated and new.

*Elicitation procedure* The following four target quantities were selected: the expected PTJ for the marginal of both factors EnC (1) and ReP (2), the expected difference in PTJ ($\Delta$PTJ) between repeated and new statements for each EnC level (3), and the expected $R^2$ defined as a variance ratio of the modeled predictive means and the predictive observations including the residual variance, $R^2= \text {var}(\theta _i) / \text {var}(y_i)$ (4). For target quantities 1-3 quantile elicitation is used and for target quantity 4 histogram elicitation. As hypothetical ground truth, we specify the following hyperparameter vector $\lambda ^* = (\mu _0=0.12, \sigma _0=0.02, \mu _1=0.15, \sigma _1=0.02, \mu _2=-0.02, \sigma _2=0.06, \mu _3=-0.03, \sigma _3=0.06, \mu _4=-0.02, \sigma _4=0.03, \mu _5=-0.04, \sigma _5=0.03, \alpha =20., \beta = 200.)$. The elicited statistics conditional on $\lambda ^*$ are depicted in Fig. 1. The first column depicts the histogram for $R^2$ and the remaining columns the results of quantile-based elicitation.

*Optimization* To instantiate the optimization process the hyperparameters $\lambda$ are randomly initialized as follows: $\mu _k \sim \text {Normal}(0, 0.1)$, $\log \sigma _k \sim \text {Uniform}(-2, -4)$, $\log \alpha \sim \text {Normal}(3, 0.1)$, and $\log \beta \sim \text {Normal}(5, 0.1)$, whereby the scale, concentration, and rate parameter are initialized on the log scale. Subsequently, we simulate from the forward model and compute the corresponding model-implied target quantities, along with the elicited statistics. The discrepancy between the model-implied and true elicited statistics can then be computed and the hyperparameters updated. The learning process is considered completed once the maximum number of epochs has been reached. Details about the optimization algorithm can be found in the "Methods" section and the corresponding specification of the algorithm parameters can be found in "Appendix B.1".

**Figure 2:**
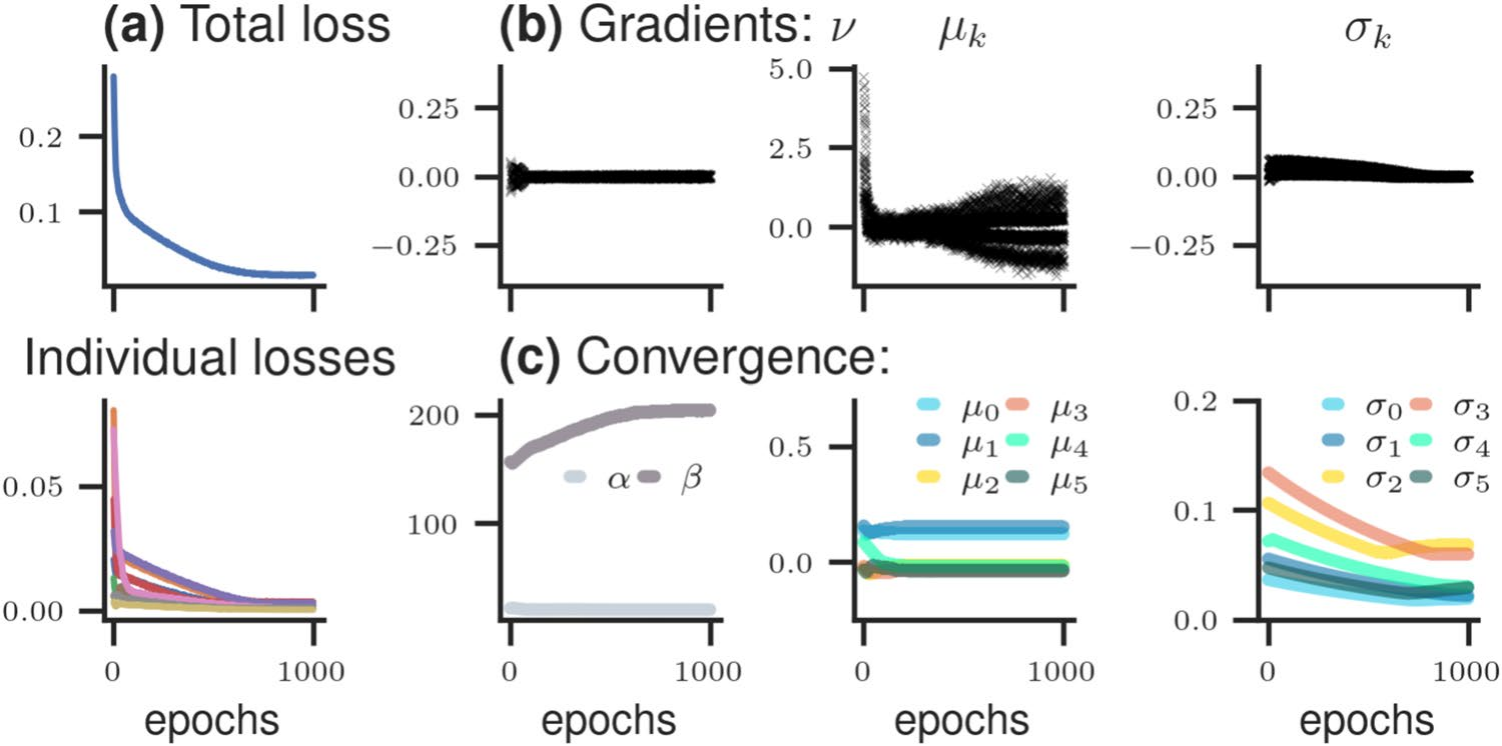
Convergence diagnostics for simulation study 1. (**a**) loss value across epochs, demonstrating the desired decreasing trend of all loss values (i.e., the total loss and the individual loss components); (**b**) expected decreasing trend towards zero of the gradients for each learned hyperparameter $\lambda$; (**c**) update values of each learned hyperparameter after each iteration step (epoch), stabilizing in the long run at a specific value.

To assess whether learning was successful, we first check the *convergence diagnostics* as summarized in Fig. 2. Examining the loss functions depicted in the leftmost column demonstrates the desired decreasing behavior for both the total loss as well as the individual loss components. The gradients of the hyperparameters $\lambda$ are depicted in the upper, right row, indicating the expected decreasing behavior towards zero across time. Finally, convergence of hyperparameters $\lambda$ during the learning process is illustrated in the lower, right row.

**Figure 3:**
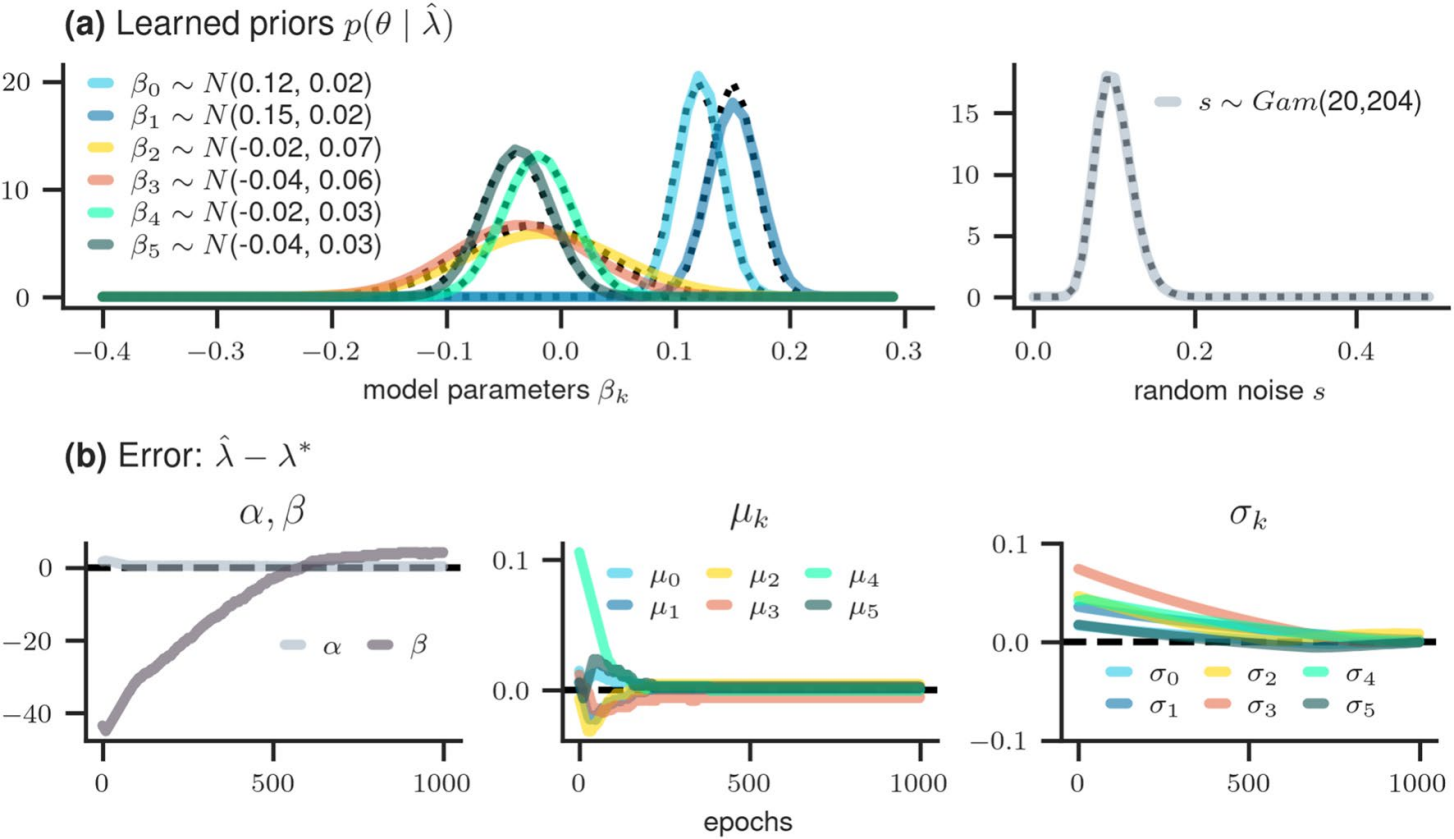
Results of simulation study 1. (**a**) true (dotted line) and learned (solid line) prior distributions per model parameter $\beta _k$ and *s*; (**b**) error between learned and true hyperparameter values $(\alpha, \beta , \mu _k, \sigma _k)$ over time.

*Results* After having confirmed successful convergence, we shift our focus to the simulation results as depicted in Fig. 3. The final learned hyperparameter $\lambda$ is computed as the average of the last 30 epochs. The resulting *learned* prior distributions are shown in the upper row of Fig. 3. Solid lines indicate the learned priors and dotted lines the *true* priors (according to $\lambda ^*$).

The substantial overlap between these distributions indicates a successful learning process. This is further emphasized in the second row, where the error between the learned and true hyperparameter values gradually decreases towards zero.

### Simulation study 2: GLMs—Binomial model

*Setup* In simulation study 2 we utilize a binomial response distribution with a logit-link function for the probability parameter. As accompanying example, we use the Haberman’s survival dataset from the UCI machine learning repository^27^. The dataset contains cases from a study on the survival of patients who had undergone surgery for breast cancer. In the following, we use the detected number of axillary lymph nodes that contain cancer (i.e., (positive) axillary nodes) as numerical predictor *X* which consists in total of 31 observations ranging between 0 and 59 axillary nodes. The dependent variable *y* is the number of patients who died within five years out of $T=100$ trials for each observation $i=1,\ldots ,N$. We consider a simple binomial regression model with one continuous predictor

2 $$\begin{aligned} y_{i}&\sim \text {Binomial}(T, \theta _i)\\ \text {logit}(\theta _i)&= \beta _0 + \beta _1 x_i \\ \beta _k&\sim \text {Normal}(\mu _k, \sigma _k) \quad \text {for} \quad k = 0,1. \end{aligned}$$

We assume normal priors for the regression coefficients, with mean $\mu _k$ and standard deviation $\sigma _k$ for $k=0,1$. Through the logit-link function, the probability $\theta _i$ is mapped to the scale of the linear predictor. The objective is to learn four hyperparameters $\lambda =(\mu _k, \sigma _k)$.

*Elicitation procedure and optimization* As target quantities we select the expected number of patients who died within five years for different numbers of axillary nodes $x_i$, with $i = 0, 5, 10, 15, 20, 25, 30$. For each selected design point, we consider quantile-based elicitation. The hypothetical ground truth is defined by the following hyperparameter vector $\lambda ^*=(\mu _0=-0.51, \sigma _0=0.06, \mu _1=0.26, \sigma _1=0.04)$. The specification of the algorithm parameters for the optimization procedure can be found in "Appendix B.2". The convergence diagnostics check follows the same procedure as discussed for simulation study 1, and showed successful convergence (see "Appendix B.2.1").

**Figure 4:**
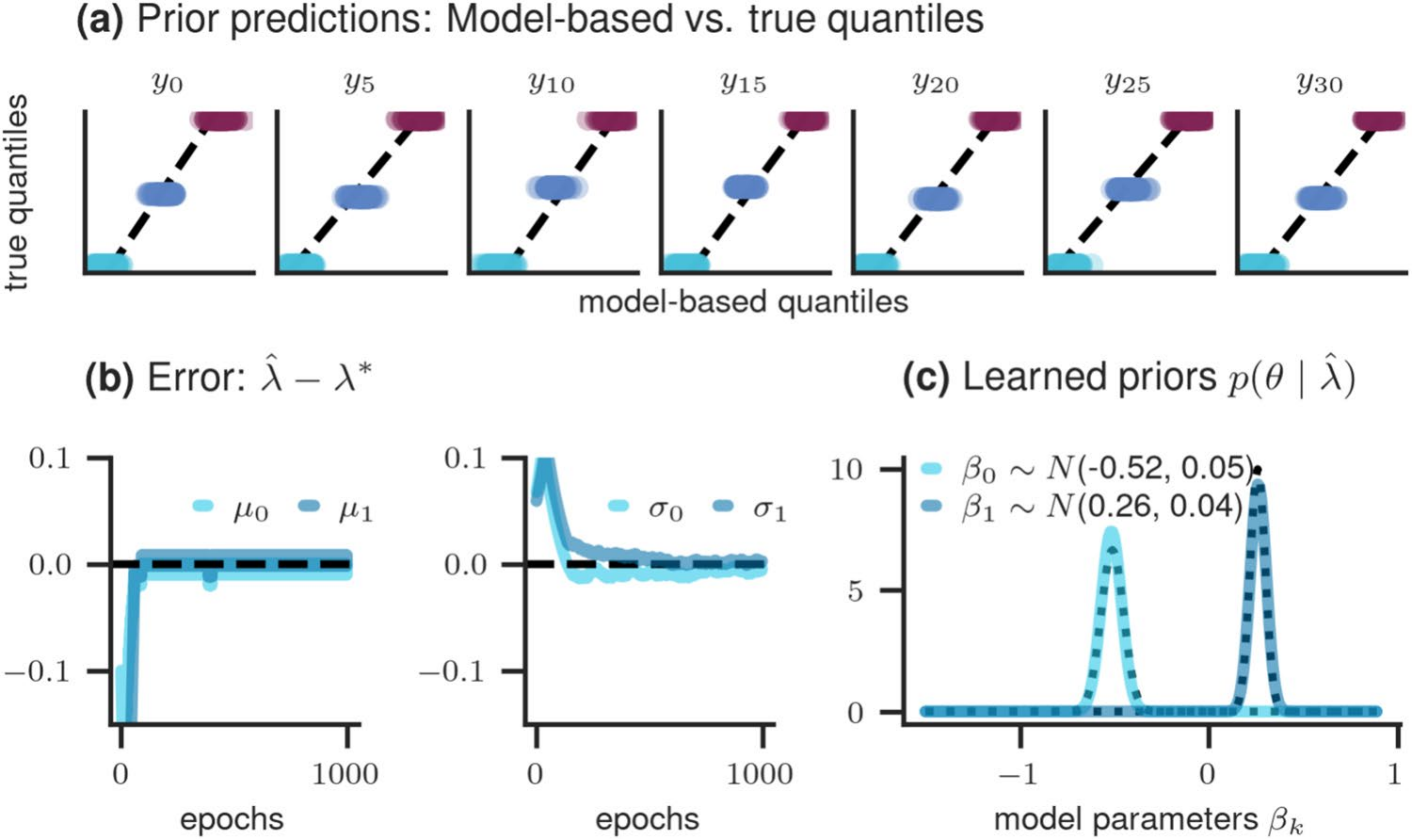
Results of simulation study 2: (**a**) comparison between learned and true quantiles for each selected $x_i$; (**b**) learning of hyperparameters across epochs, showcasing the difference between the true and learned values; (**c**) true (dotted line) and learned (solid line) prior distributions of each model parameter.

*Results* The simulation results, based on the final learned hyperparameters $\lambda$, are presented in Fig. 4.

The upper row shows a comparison between the true and learned quantiles for each number of axillary nodes $x_i$, revealing an almost perfect match between both quantities. In the lower right panels, the error between the true and learned hyperparameters is depicted and indicates successful learning. Additionally, the lower right panel presents the true (dotted line) and learned (solid line) prior distributions which show a perfect match.

### Simulation study 3: GLMs—Poisson model

*Setup* In simulation study 3, we expand our examination of count data likelihoods to include a Poisson distribution. For demonstration purposes, we adapt an example from Johnson et al.^28^, which investigates the number of LGBTQ+ anti-discrimination laws in each US state. The distribution of these laws is assumed to follow a Poisson distribution, with the rate parameter being influenced by demographic and voting trend. The demographic trend is quantified by the percentage of a state’s residents living in urban areas, ranging from $38.7\%$ to $94.7\%$. Additionally, the voting trend is represented by historical voting patterns in presidential elections, categorizing each state as consistently voting for the Democratic or Republican candidate or being a Swing state. We employ a Poisson regression model including one treatment-coded categorical predictor: the *voting trend*. This predictor has three levels: Democrats, Republicans, and Swing, with Democrats serving as the reference category. Furthermore, the model incorporates one continuous predictor: the *demographic trend*, measured as a percentage. The Poisson regression model is represented as follows

3 $$\begin{aligned} y_i&\sim \text {Poisson}(\theta _i)\\ \log (\theta _i)&= \beta _0 + \beta _1 x_1 + \beta _2 x_2 + \beta _3 x_3\\ \beta _k&\sim \text {Normal}(\mu _k, \sigma _k) \quad \text { for } k=0,\ldots ,3. \end{aligned}$$

Here, $y_i$ is the number of counts for observation $i = 1, \ldots , N$. The counts follow a Poisson distribution with rate $\theta _i$ and log-link function. The rate parameter is predicted by a linear combination of the two predictors demographic and voting trend. All regression coefficients are assumed to have normal prior distributions with mean $\mu _k$ and standard deviation $\sigma _k$ for $k=0,\ldots ,3$. Our goal is to learn eight hyperparameters $\lambda =(\mu _k, \sigma _k)$.

*Elicitation procedure* We consider two target quantities: the predictive distribution of the group means for states categorized as Democrats, Republicans, and Swing, and the expected number of LGBTQ+ anti-discrimination laws for selected US states $x_i$ with $i=0, 13, 14, 35, 37, 48$. Quantile-based elicitation is used for the distribution of group means and histogram elicitation for the observations per US state. Furthermore, the expected maximum number of LGBTQ+ anti-discrimination laws in one US state is required. This value is used as upper truncation threshold, $t^u$, of the Poisson distribution which is needed for applying the Softmax-Gumbel Trick that allows for computing gradients for discrete random variables (see Section "Gradient-based optimization" for details). For the current example, we assume $t^u=80$ and define the following hyperparameter vector $\lambda ^*$ representing the ground truth: $\lambda ^*=(\mu _0= 2.91, \sigma _0= 0.07, \mu _1= 0.23, \sigma _1= 0.05, \mu _2= -1.51, \sigma _2= 0.135, \mu _3= -0.61, \sigma _3= 0.105)$. The specification of algorithm parameters for the optimization procedure as well as a figure summarizing the convergence diagnostics can be found in "Appendix B.3".

**Figure 5:**
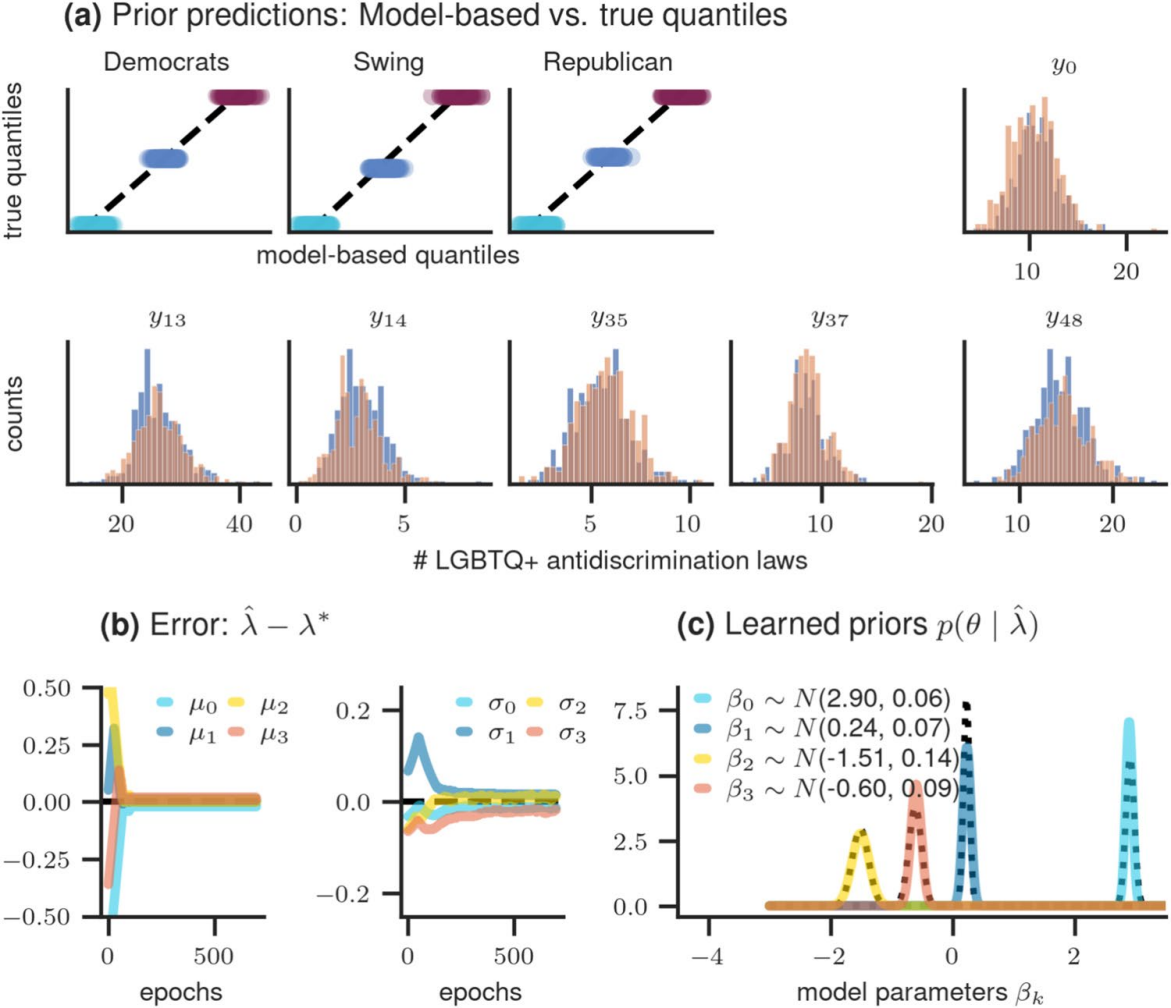
Results of simulation study 3: (**a**) comparison between model-based and true elicited statistics. First three panels depict quantile-based elicitation for the group means, while the remaining upper panels show histogram elicitation for each state $x_i$. The model-based histograms are depicted in blue and the ground truth in red. (**b**) learning of hyperparameters across epochs, showcasing the difference between the true and learned values; (**a**) true (dotted line) and learned (solid line) prior distributions of the model parameters.

*Results* The learned hyperparameters’ results are presented in Fig. 5. In the upper panels, a comparison between model-based and true elicited statistics is presented and shows a high level of agreement: quantile-based elicitation for the voting groups is depicted in the first three panels and histogram elicitation for single states in the remaining upper panels.

The model-based histograms are depicted in blue and the ground truth in red. The lower left panels demonstrate that the error between learned and true hyperparameter values converges towards zero over time. Finally, the learned prior distributions are depicted in the lower right panel, with solid lines representing the learned and dotted lines the true priors.

### Simulation study 4: Hierarchical model

*Setup* In this concluding simulation study, we investigate the performance of our elicitation method when applied to a hierarchical model. This specific model class poses a distinct challenge for analysts and domain experts alike due to the inherent complexity of the model and the non-intuitive nature of varying effects (i.e., varying intercepts and slopes). Our method allows for learning prior distributions within a hierarchical framework, while relying on expert knowledge that is articulated in terms of interpretable target quantities.

The accompanying example draws inspiration from the *sleepstudy* dataset^29^. This dataset contains information about the average reaction time (RT) in milliseconds for *N* individuals who undergo sleep deprivation for nine consecutive nights. In order to construct a model for this data, we consider a hierarchical model with days serving as a continuous predictor *x*,

4 $$\begin{aligned} y_{ij}&= \text {Normal}(\theta _{ij}, s)\\ \theta _{ij}&= \beta _0 + u_{0,j} + (\beta _1 + u_{1,j}) x_{ij} \\ ( u_{0,j}, u_{1,j} )&\sim \text {MvNormal}\left( {\textbf {0}}, \Sigma _u\right) \\ \Sigma _u&= \begin{pmatrix}\tau _0^2 &{} \rho _{01} \tau _0 \tau _1 \\ \rho _{01} \tau _0 \tau _1 &{} \tau _1^2\end{pmatrix} \\ \beta _k&\sim \text {Normal}(\mu _k, \sigma _k) \qquad \text { for } k=0,1\\ \tau _k&\sim \text {TruncatedNormal}(0, \omega _k) \qquad \text {for } k=0,1\\ \rho _{01}&\sim \text {LKJ}(\alpha _\text {LKJ}) \\ s&\sim \text {Gamma}(\alpha , \beta ). \end{aligned}$$

Here $y_{ij}$ represents the average RT for the $j^{th}$ participant at the $i^{th}$ day with $j=1,\ldots , 200$ and $i=0,\ldots ,9$. The RT data is assumed to follow a normal distribution with local mean $\theta _{ij}$ and within-person standard deviation *s*. Here, $\theta _{ij}$ is predicted by a linear combination of the continuous predictor *x* with overall slope $\beta _1$ and intercept $\beta _0$. Given the potential variation in both baseline and change in RT across participants, the model incorporates varying (i.e., “random”) intercepts $u_{0,j}$ and slopes $u_{1,j}$. These varying effects follow a multivariate normal distribution, centered at a mean vector of zero and with a covariance matrix $\Sigma _u$. This encodes the variability ($\tau _0, \tau _1$) and the correlation ($\rho _{01}$) between $u_{0,j}$ and $u_{1,j}$. For the resulting set of model parameters, the following prior distributions are assumed: A normal distribution for the overall (i.e., “fixed”) effects $\beta _k$ ($k = 0, 1$) with mean $\mu _k$ and standard deviation $\sigma _k$. A truncated normal distribution centered at zero with a standard deviation of $\omega _k$, is employed for the person-specific variation $\tau _k$, which is constrained to be positive. The correlation parameter $\rho _{01}$ follows a Lewandowski-Kurowicka-Joe [LKJ;^30^] distribution with scale parameter $\alpha _\text {LKJ}$. In the subsequent context, we set $\alpha _\text {LKJ}$ to 1. Additionally, a Gamma prior distribution with concentration $\alpha$ and rate $\beta$ is used for the within-person (error) standard deviation *s*. The goal is to learn eight hyperparameters $\lambda =(\mu _k,\sigma _k, \omega _k, \alpha , \beta )$.

*Elicitation procedure and optimization* We consider the following target quantities and elicitation techniques: quantile-based elicitation for the expected average RT for specific days $x_i$, where $i = 0,2,5,6,9$. Moment-based elicitation using mean and standard deviation for the within-person standard deviation *s* (elicitation in the parameter space), and histogram-elicitation for the expected distribution of $R^2$ for the initial and final day ($i = 0,9$). We define the expected ground truth by the following hyperparameter vector $\lambda ^*=(\mu _0=250.40, \mu _1=30.26, \sigma _0=7.27, \sigma _1=4.82, \omega _0=33.00, \omega _1=23.00, \alpha =200, \beta =8)$. Please refer to "Appendix B.4" for detailed information about the algorithm parameters of the optimization procedure together with a figure summarizing the convergence diagnostics indicating successful convergence.

**Figure 6:**
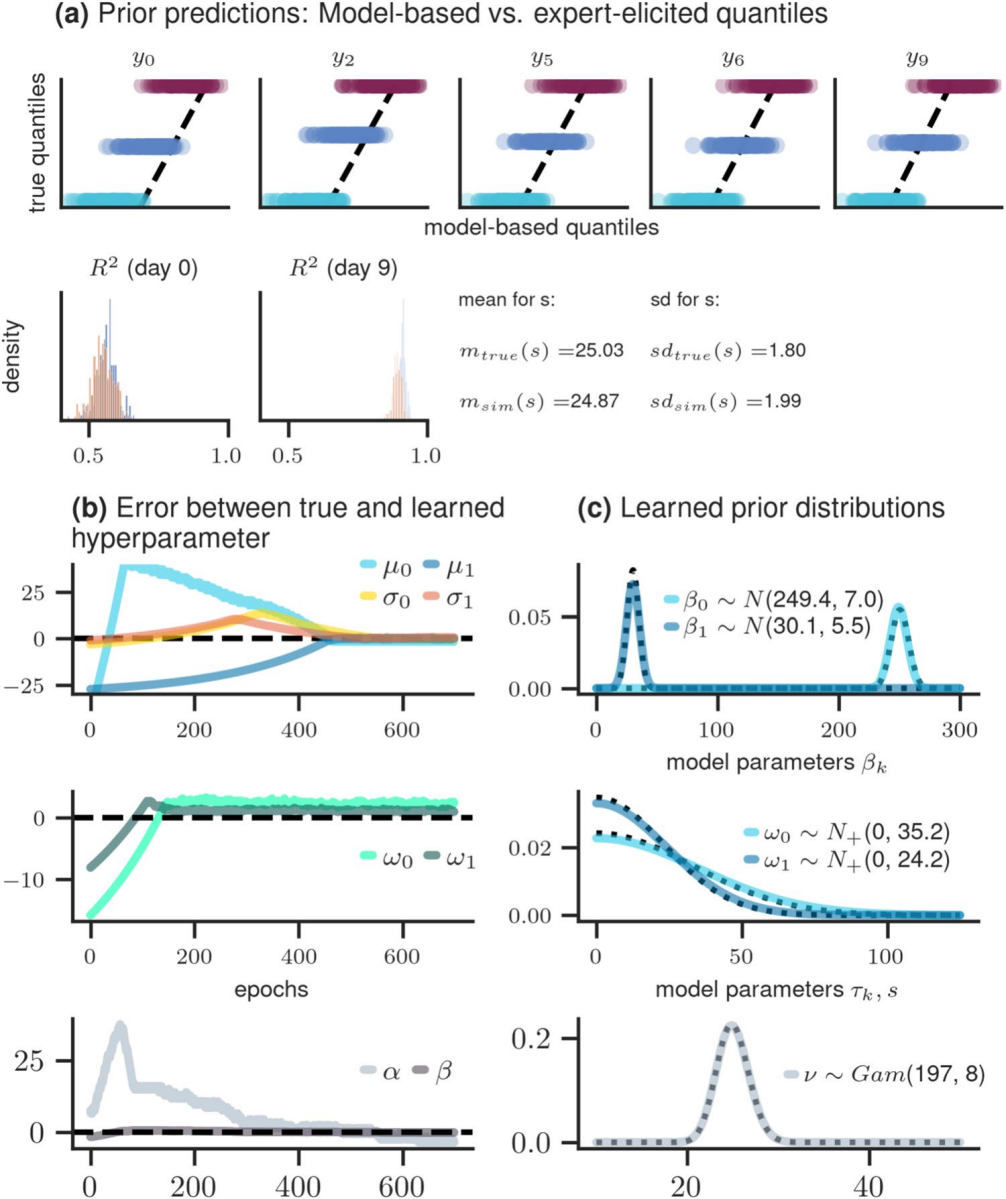
Results of simulation study 4: (**a**) comparison between model-based and true elicited statistics. First row depicts quantile-based elicitation for each day $x_i$. Second row shows histogram-based elicitation for $R^2$ (red true and blue model-implied) and moment-based elicitation for model parameter *s* ($m_\text {true}, sd_\text {true}$ stands for true elicited mean and standard deviation, respectively). (**b**) learning of hyperparameters across epochs, showcasing the difference between the true and learned values; (**c**) true (dotted line) and learned (solid line) prior distributions of each model parameter.

*Results* Figure 6 presents the results derived from the optimization process. The upper two rows depict the congruence between simulation-based and true elicited statistics, effectively highlighting successful learning. The first row illustrates the alignment between true and learned quantiles for the chosen days $x_i$. The first two plots in the lower row show the distributions of $R^2$ as predicted by the model and the ground truth for day 0 and 9. The model-based histograms are depicted in blue and the ground truth in red. Finally, moment-based elicitation (i.e., mean and standard deviation) for the model parameter *s* is depicted as remaining information in the second row.

The learned prior distributions for each model parameter are depicted in the lower, right column of Fig. 6. The high overlap between true (dashed lines) and learned (solid lines) prior distributions indicates an additional instance of successful learning. This is further supported by the assessment of the error between true and learned hyperparameters in the lower left column, revealing a progressive convergence towards zero across epochs.

## Discussion

When developing Bayesian models, analysts face the challenge of specifying appropriate prior distributions for each model parameter, involving both the choice of the distributional family as well as the corresponding hyperparameter values. We proposed an elicitation method that assists analysts in identifying the hyperparameter values of given prior distribution families based on expert knowledge. Our method accommodates various types and formats of expert knowledge and is agnostic to the specific probabilistic model. In our simulation studies, we demonstrated the excellent performance of our method for various modeling tasks and kinds of expert knowledge. Despite these highly promising results, some relevant limitations remain, which are discussed below together with ideas for future research.

Our method employs gradient-based optimization to learn hyperparameter values which requires only the ability to sample from the generative model. However, it comes with the prerequisite that all operations and functions in the computational graph are differentiable or admit a reparameterization whose gradients can be approximated with sufficient accuracy. Consequently, for discrete random variables, specific techniques, such as the Softmax-Gumbel trick, are necessary. Alternatively, one could opt for optimization methods that entirely forego gradient computations such as Bayesian optimization^31^ as used by Manderson & Goudie^6^. Nevertheless, this choice has its own limitations, notably in terms of scalability to higher-dimensional spaces^32^.

Having a suitable optimization method is fundamental for learning hyperparameters based on expert knowledge. However, there are cases where hyperparameters cannot be uniquely determined from available expert data, leading to different learned hyperparameters upon multiple replications of the learning process. This situation raises the question of how to choose between prior distributions that represent the elicited expert knowledge equally well. Initial approaches, such as incorporating a regularization term in the loss function to favor priors with higher entropy, have been proposed to address this challenge^6^. Another avenue to achieve model identification involves the model architecture. For instance, statistical models that adopt joint priors for their parameters and thus keep the number of hyperparameters low, are expected to exhibit improved model identification [e.g.,^33^]. Nevertheless, further research is needed to develop informative metrics for assessing model identification as well as techniques that can efficiently handle unidentified models^34^.

Finally, all gradient-based optimization methods share the objective of finding an optimal *point estimate* for the hyperparameters $\lambda$. By adopting this approach, any uncertainties surrounding the value of $\lambda$ are neglected, despite the potential introduction of uncertainty during the prior elicitation process. To address this limitation, it would be advantageous to adopt a probabilistic approach that explicitly accounts for uncertainty in the hyperparameters [e.g.,^5,16^]. Given the flexibility of our method, it can readily accommodate this concept, offering a promising avenue for future development and next steps.

## Methods

We propose a new elicitation method for translating knowledge from a domain expert into an appropriate parametric prior distribution. Building on recent contributions^6,9,16^ we developed a model-agnostic method in which the search for appropriate prior distributions is formulated as an optimization problem. Thus, the objective is to determine the optimal hyperparameters that minimize the discrepancy between model-implied and expert-elicited statistics. Our elicitation method supports expert feedback in both the space of parameters and observable quantities (i.e., a *hybrid* approach) and minimizes human effort. The key ideas underlying our method are outlined as follows:

1. The analyst defines a generative model comprising a likelihood function $p(y\mid \theta )$ and a parametric prior distribution $p(\theta \mid \lambda )$ for the model parameters, where $\lambda$ represents the prior hyperparameters to be inferred from expert knowledge.
2. The analyst selects a set of target quantities, which may involve queries related to observable quantities (data), model parameters, or anything else in between.
3. The domain expert is queried using a specific elicitation technique for each target quantity (*expert-elicited statistics*) .
4. From the generative model, parameters and (prior) predictive data are simulated, and the predefined set of target quantities is computed (*model-implied quantities*) .
5. The discrepancy between the model-implied and the expert-elicited statistics is evaluated via a specific loss function.
6. Stochastic gradient descent is employed to update the hyperparameters $\lambda$ so as to minimize the loss function.
7. Steps 4 to 6 are repeated iteratively until an optimal set of hyperparameters $\lambda$ is found that minimizes the discrepancy between the model-implied and the expert-elicited statistics.

**Figure 7:**
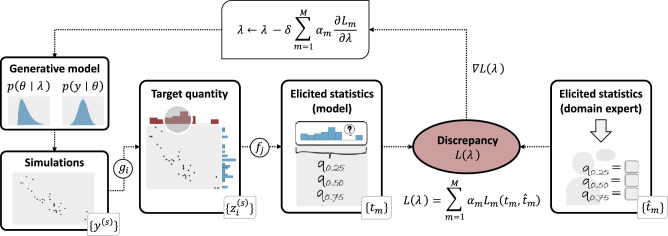
Graphical illustration of our simulation-based elicitation method. Step 1 involves employing elicitation techniques to extract target quantities from the domain expert. Subsequently, the objective is to minimize the discrepancy between model-implied and expert-elicited statistics by optimizing the hyperparameters $\lambda$. The optimization process iteratively simulates data using the current hyperparameters $\lambda$, computes model-implied elicited statistics, compares them with the expert-elicited statistics using a loss function ($L_m$), and updates $\lambda$ to improve agreement between model-implied and expert-elicited statistics. Here, $\alpha _m$ is the weight of the $m^{th}$ loss component and $\delta$ is the step size.

In the upcoming sections, we will delve into the details of the outlined approach. To provide a visual representation of all steps involved in our proposed elicitation method, Fig. 7 presents a graphical overview. In addition, readers can find a symbol glossary in "Appendix A" for a quick reference. An illustrative example that details each step of the workflow using specific values can be found in our online supplement https://osf.io/rxgv2.

### Elicited statistics from the expert

We assume that the analyst queries the domain expert regarding a predetermined set of *I* target quantities, represented as $\{z_i\} := \{z_i\}_{i=1}^I$. The set $\{z_i\}$ is selected by the analyst depending on the requirements of the statistical model and the knowledge of the expert^5,9,21^. Once this set is defined, the expert is queried regarding each individual target quantity $z_i$, assuming that the expert possesses an implicit representation, denoted as ${\hat{z}}_i$, which can be accessed using expert *elicitation techniques*^6,14,16,35^. While numerous elicitation techniques have been proposed in the literature^21^, it can be argued that these techniques essentially represent different facets of the following three general method families: moment-based elicitation (e.g., mean and standard deviation), quantile-based elicitation (e.g., median, lower quartile, and upper quartile), and histogram elicitation (e.g., constructing a histogram by sampling from the distribution of $z_i$). Each target quantity $z_i$ can be elicited through a distinct elicitation technique $f_j$. Within our notation, we represent the $i^{th}$ target quantity elicited from the expert through the $j^{th}$ elicitation technique as ${\hat{t}}_m = {\hat{t}}_{ij}$ and refer to it as *elicited statistics* ${\hat{t}}_m = f_j({\hat{z}}_i)$. The index $m = 1, \ldots , M$ indicates the number of elicited statistics resulting from specific target-quantity $\times$ elicitation-technique combinations, as selected by the analyst.

### Model-based quantities

Considering the set of elicited statistics queried from the expert $\{{\hat{t}}_m\}$, it is possible to assess the extent to which a generative model, as specified by the analyst, aligns with the expert’s expectations. A Bayesian model comprises a likelihood $p(y \mid \theta )$ as well as parametric prior distributions $p(\theta \mid \lambda )$ for the model parameters $\theta$. Here, $\lambda$ represents the prior hyperparameters to be inferred by our method and *y* a vector of observations. The degree to which the model captures the expert’s expectations relies on the specific values assigned to $\lambda$. Consequently, the objective is to identify a specification of $\lambda$ that minimizes the discrepancy between the set of expert-elicited statistics $\{{\hat{t}}_m\}$ and model-implied elicited statistics, $\{t_m\}$.

First, we need to derive the set of model-implied target quantities $\{z_i\}$. As a target quantity can represent an observable, a parameter, or anything else in between, we define it in the most general form as a function of the model parameters $\theta$, denoted as $z_i = g_i(\theta )$, where the function *g* can take on various forms and be of deterministic or stochastic nature. In its simplest form, the target quantity directly corresponds to a parameter of interest in the data-generating model ($z_i=g_i(\theta ) = \theta _i$; i.e., *g* would be a simple projection). Alternatively, *g* can be aligned with the generative model of the data, resulting in the target quantity being equivalent to the observations ($z_i = g_i(\theta ) = y$). Moreover, the function *g* can take on more complex forms. Suppose the domain expert provides prior knowledge about the coefficient of determination $R^2$ commonly used to measure model fit in regression models^36^. To obtain the corresponding model-implied $R^2$, we first generate observations *y* using the specified generative model and then compute the $R^2$ value from the observations. Given the set of model-implied target quantities, we get the respective model-implied *elicited statistics*, denoted by $\{t_m\}$, by applying the elicitation technique $f_j$ to the target quantity $z_i: t_m = f_j(z_i)$.

A challenge with this approach is that the distribution of $\{t_m\}$ may not be analytical or have a straightforward computational solution. For instance, consider the case where the target quantity is equivalent to the observations, $z_i = y$. In this case, the distribution of the predicted observations *y* gives rise to an integral equation known as the prior predictive distribution (PPD), denoted by $p(y \mid \lambda )$ and defined by averaging out the prior from the generative model: $p(y \mid \lambda ) = \int _\Theta p(y \mid \theta ) p(\theta \mid \lambda ) d\theta .$ Obtaining a closed-form expression for this integral is only feasible in certain special cases, such as when dealing with conjugate priors. This challenge extends to all situations where the target quantity is a function of the observations *y*. However, to ensure the broad applicability of our elicitation method to a wide range of models, we adopt a simulation-based approach that relies solely on the ability to generate samples from the relevant quantities. Bayesian models, by their very formulation, can simulate data from their prior and likelihood distributions, thereby enabling us to generate samples from the Bayesian probabilistic model^2,37^. For example, in the case where $z_i = y$, the simulation-based procedure involves two steps: Firstly, we sample the model parameters from the prior distribution conditioned on hyperparameters $\lambda$: $\theta ^{(s)} \sim p(\theta \mid \lambda )$. Subsequently, we generate data by sampling from the likelihood distribution, resulting in $y^{(s)} \sim p(y \mid \theta ^{(s)})$. The superscript (*s*) is used to denote the $s{\rm th}$ sample of the corresponding simulated quantity. By repeating these steps, we can generate a collection of *S* simulations $\{y^{(s)}\}:= \{y^{(s)}\}_{s=1}^S$, where each element corresponds to a data point drawn from the PPD: $y^{(s)} \sim p(y\mid \lambda ).$

### Multi-objective optimization problem

Once the elicited statistics $\{{\hat{t}}_m\}$ from the expert and a procedure to compute the corresponding model-implied elicited statistics $\{t_m\}$ are chosen, the focus can be shifted towards the main objective: Determine the hyperparameters $\lambda$ that minimize some discrepancy measure (loss function) $L(\lambda )$ between the expert-elicited $\{{\hat{t}}_m\}$ and the model-implied statistics $\{t_m\}$ = $\{t_m(\lambda )\}$. Since the evaluation of the discrepancy extends to all elicited statistics $\{t_m\}$, $L(\lambda )$ has to be formulated as a multi-objective loss function. This loss function encompasses a linear combination of discrepancy measures $L_m$, with corresponding weights $\alpha _m$ (see Section "Dynamic weight averaging"). In the following, we will also use the term *loss components* to refer to the individual components in the weighted sum. The selection of the discrepancy measure $L_m$ is contingent upon the elicited statistic, therefore different choices may be appropriate depending on the specific quantity to be compared (see Section "Maximum mean discrepancy"). Independently of these specific choices, our main objective can be written as

5 $$\begin{aligned} \lambda ^* = \arg \min _{\lambda } L(\lambda ) = \arg \min _{\lambda } \sum _{m=1}^M\alpha _{m} L_m(t_m(\lambda ), {\hat{t}}_m), \end{aligned}$$

where $\lambda ^*$ denotes the optimal value of the hyperparameters $\lambda$ given the provided expert knowledge.

### Gradient-based optimization

The optimization procedure for solving Eq. (5) follows an iterative approach. In each iteration, we sample from the generative model, compute the model-implied elicited statistics, and update the hyperparameters $\lambda$. This update relies on calculating the gradient of the discrepancy loss with respect to the hyperparameters $\lambda$ and adjusting them in the opposite direction of the gradient^38^. The procedure continues until a convergence criterion is met, usually when all elements of the gradient approach zero. We employ mini-batch stochastic gradient descent (SGD) with automatic differentiation, facilitated by the reparameterization trick [explicit or implicit;^39,40^. In our case, stochasticity in mini-batch SGD arises naturally as we simulate new model-implied quantities at each iteration step.

The reparameterization trick involves splitting the representation of a random variable into stochastic and deterministic parts. By differentiating through the deterministic part, we can compute gradients with respect to $\lambda$ using automatic differentiation^41^. To leverage backpropagation, it is essential that all operations and functions in the computational graph are differentiable with respect to $\lambda$. This requirement extends to the loss function and all computational operations in the generative model^9,16^.

However, dealing with discrete random variables poses a challenge due to the non-differentiable nature of discrete probability distributions, making gradient descent through such variables difficult. One approach to overcome this challenge is to use continuous relaxation of discrete random variables, which enables the estimation of gradients and thus the use of gradient-based optimization methods for models that involve discrete random variables^42–44^. For instance, both Maddison et al.^43^ and Jang et al.^44^ independently proposed the Gumbel-Softmax trick, which approximates a categorical distribution, with finite number of categories, with a continuous distribution. Joo et al.^45^ proposed an extension of the Gumbel-Softmax trick to arbitrary discrete distributions by introducing truncation for those distributions that lack upper and/or lower boundaries. We used the Softmax-Gumbel trick in simulation study 2 and applied the truncation technique in simulation study 3 (Sections "Simulation study 2: GLMs—binomial model" and "Simulation study 3: GLMs—Poisson model").

### Maximum mean discrepancy

A key aspect of the optimization problem, as expressed in Eq. (5), is the selection of an appropriate discrepancy measure, $L_m$. This measure depends on the characteristics of the elicited statistics $\{t_m\}$ and $\{{\hat{t}}_m\}$. Given that our method entails the generation of $\{t_m\}$ through repeated sampling from the generative model, a loss function is needed that can quantify the discrepancy between samples. The *maximum mean discrepancy* (MMD)^46,47^ is a kernel-based method designed for comparing two probability distributions when only samples are available, making it suitable for our specific requirements. We utilize the MMD for all loss components in our applications. This decision is based on the robust simulation results and excellent performance as reported in the simulation studies section. That said, our method does not strictly require the MMD, but allows analysts to choose a different discrepancy measure for each loss component, if desired.

Let $x= \{x_1, \ldots , x_n \}$ and $y= \{y_1, \ldots , y_m \}$ be iid draws from the distributions *p* and *q*, respectively. The MMD measures the distance between two sets of samples by taking the maximum difference in sample averages over a function class ${\mathcal {F}}$ (Def. 2)^46^: $\text {MMD} = \sup _{f \in {\mathcal {F}}}\left( {\mathbb {E}}_{x\sim p}[f(x)] - {\mathbb {E}}_{y\sim q}[f(y)] \right) .$ If ${\mathcal {F}}$ is a unit ball in the universal *reproducing kernel Hilbert space* ${\mathcal {H}}$ with associated reproducing kernel $k(\cdot ,\cdot )$, the MMD is a strictly proper divergence, thus equals zero if and only if $p=q$^47^. The (biased) empirical estimate of the squared-MMD is defined as $\text {MMD}^2_b = \frac{1}{n^2}\sum _{i,j = 1}^n k(x_i, x_j) + \frac{1}{m^2}\sum _{i,j = 1}^m k(y_i,y_j) - \frac{2}{mn} \sum _{i,j=1}^{m,n} k(x_i,y_j)$ where $k(\cdot , \cdot )$ is a continuous and characteristic kernel function. In our simulations, we used the *energy distance* kernel $k(x,y)= - ||x-y||$, as proposed by Feydy^48^ and Feydy et al.^49^, which does not require an extra hyperparameter for tuning.

### Dynamic weight averaging

In addition to selecting an appropriate discrepancy measure, another important consideration involves choosing the weights $\alpha _m$ in Eq. (5). One possibility is for the user to customize the choice of $\alpha _m$, signifying the varying degrees of importance for each loss component in a particular application^50^. However, another consideration refers to the *task balancing problem*. When employing stochastic gradient descent to minimize the objective as outlined in Eq. (5), the hyperparameters $\lambda$ are updated according to the following rule $\lambda \leftarrow \lambda - \delta \sum _{m=1}^M \alpha _m \frac{\partial L_m}{\partial \lambda },$ where $\delta$ is the step size (i.e., learning rate). The equation suggests that the hyperparameter update may not yield optimal results if one loss component significantly outweighs the others^50^.

Consequently, a strategy is needed to dynamically modify the weights $\alpha _m$ to ensure effective learning of all loss components. For example, the *dynamic weight averaging* (DWA) method proposed by Liu et al.^51^ determines the weights based on the learning speed of each component, aiming to achieve a more balanced learning process. Specifically, the weight of a component exhibiting a slower learning speed is increased, while it is decreased for faster learning components^52^.

In our simulation studies, we consider an equal-weighting scheme ($\alpha _m=1$), as this choice has demonstrated good learning outcomes without introducing additional free hyperparameters required by most task balancing approaches. However, we believe that investigating different task balancing approaches is a promising avenue for future research. Such exploration could have a beneficial impact on the method’s performance, particularly in cases involving conflicting expert information.

### Electronic supplementary material

Below is the link to the electronic supplementary material.

Supplementary Information 1.

## Data Availability

All code and data is openly available on OSF https://osf.io/rxgv2 and GitHub https://github.com/florence-bockting/PriorLearning.
