# Supplementary material for: Simulation-based prior knowledge elicitation for parametric Bayesian models
Source: Sci Rep. 2024 Jul 27;14:17330. doi: 10.1038/s41598-024-68090-7 (PMC11283489; doi:10.1038/s41598-024-68090-7)
Supplement: Supplementary file 1 — Supplementary Information 1. [file 41598_2024_68090_MOESM1_ESM.pdf]

Appendix

A Method

A.1 Symbol Glossary

Table A.1.1: Overview of model and algorithm (hyper-)parameters

Workflow task	Notation	Label	Comment
General	seed	seed	
Generative Model	λ	model hyperparameter	hyperparameter of prior distributions
	θ	model parameter	model parameter
Gumbel-Softmax Trick	τ	temperature	$\tau = 1.0$
Gradient Descent	E	epochs / iterations	number of iterations used for learning the model hyperparameter
	B	batch size	number of simulations within one epoch; fix for all case studies
	S	prior-samples	number of samples from the prior distribution of each model-parameter within one batch
Adam Optimizer (with cosine decay schedule with restarts)	lr_0	initial learning rate	learning rate of the initial iterations
	decay-steps	decay steps	number of steps to decay over

B Simulation Studies: Convergence diagnostics and Learning Results

B.1 Simulation Study 2: LM — Normal linear regression model

To instantiate the optimization process the hyperparameter λ are randomly initialized: $\mu_k \sim \text{Normal}(0, 0.1)$, $\log \sigma_k \sim \text{Uniform}(-2, -4)$, $\log \alpha \sim \text{Normal}(3, 0.1)$, and $\log \beta \sim \text{Normal}(5, 0.1)$. The algorithm parameters for the optimization procedure were set as follows: $B = 2^7$, $E = 1000$, $S = 300$, seed = 34765771 and a cosine decay schedule with restarts with an initial learning rate of $\phi^0 = 0.001$ and first decay steps of 50. In total the learning algorithm need 99:51 minutes to finish on a GPU machine (NVIDIA Tesla K40). Figure with convergence diagnostics of the optimization results can be found in the Simulation Studies section in the main text.

B.2 Simulation Study 2: GLMs — Binomial model

To setup the learning algorithm, the following algorithm parameters are used: $B = 2^7$, $E = 1000$, $S = 300$, seed= 34764831, and a cosine decay schedule with restarts with an initial learning rate of $\phi^0 = 0.01$ and first decay steps of 50. To instantiate the optimization process the hyperparameter λ are randomly initialized: $\mu_k \sim \text{Normal}(0, 1)$ and $\log \sigma_k \sim \text{Uniform}(-2, -3)$. To improve gradient-based learning, we z-transformed the continuous predictor. The learning algorithm needed 28.85 minutes to finish on a GPU machine (NVIDIA Tesla K40). Figure B.2.1 depicts the convergence diagnostics of the optimization results.

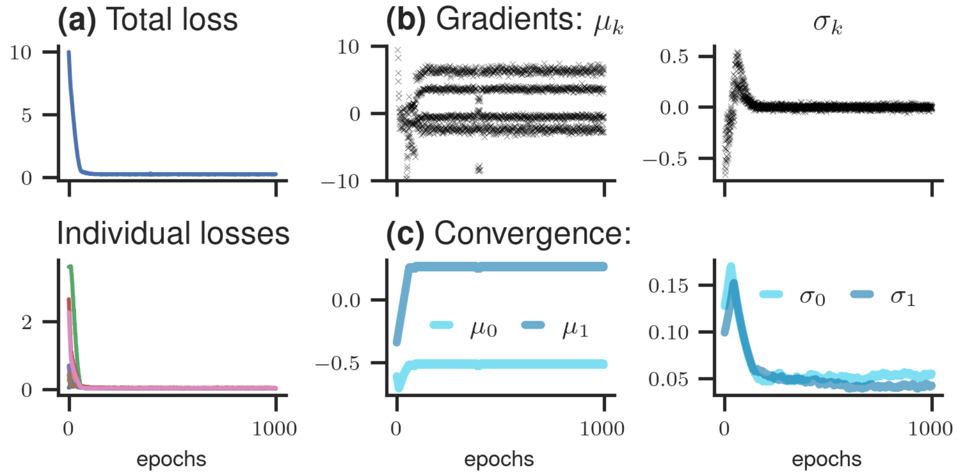

Figure B.2.1: *Convergence diagnostics Binomial model.* **(a)** loss value across epochs, demonstrating the desired decreasing trend of all loss values (i.e., total loss as well as individual loss components); **(b)** gradients of the hyperparameters λ; **(c)** learning of each hyperparameter across epochs, stabilizing in the long run at a specific value.

B.3 Simulation Study 3: GLMs — Poisson model

For the learning algorithm, we standardize (z-transform) the continuous predictor to improve learning and we set the algorithm parameters as follows: seed = 34765558, $B = 2^7, E = 700$, $S = 300$, and a cosine decay schedule with restarts, an initial learning rate of $\phi^0 = 0.01$ and first decay steps of 50 To instantiate the optimization process the hyperparameter λ are randomly initialized: $\mu_0 \sim \text{Uniform}(1, 2.5)$, $\mu_1 \sim \text{Uniform}(0, 0.5)$, $\mu_2 \sim \text{Uniform}(-1, -1.5)$, $\mu_3 \sim \text{Uniform}(-0.5, -1)$, and $\log \sigma_k \sim \text{Uniform}(-2, -5)$. The learning algorithm needed 90:92 minutes on a GPU machine (NVIDIA Tesla K40) to finish. The convergence diagnostics inspection shows successful convergence. Figure B.3.1 depicts the convergence diagnostics of the optimization results.

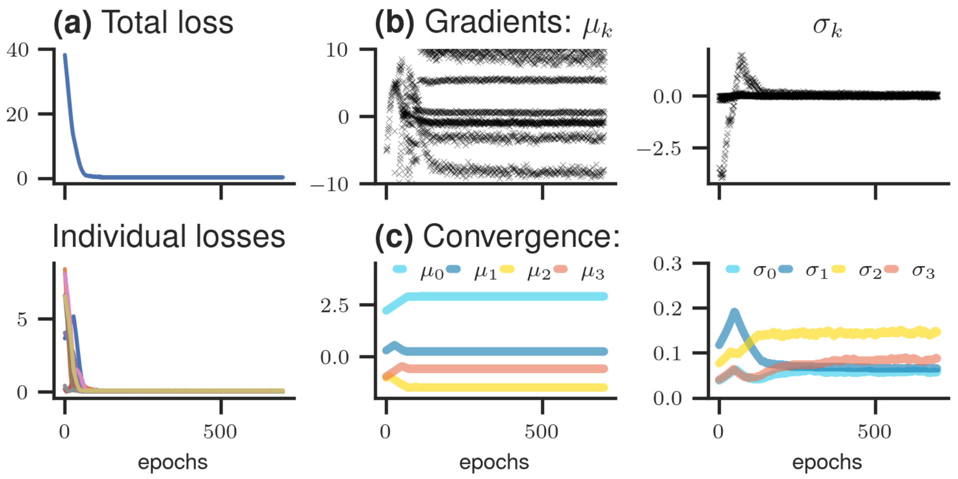

Figure B.3.1: *Convergence diagnostics for Poisson model.* **(a)** decreasing trend of the loss across epochs (i.e., upper panel total loss and lower panel individual loss components); **(b)** gradients of the hyperparameters λ across epochs; **(c)** updating of each hyperparameter across epochs.

B.4 Simulation Study 4: Hierarchical model — Normal likelihood

For the learning algorithm, we divide the continuous predictor by its standard deviation and use this scaled predictor in the optimization process. We set the algorithm parameters as follows: seed = 34765522, $B = 2^7, E = 700, S = 200$, a cosine decay schedule with restarts, an initial learning rate of $\phi^0 = 0.005$ and first decay steps of 100. To instantiate the optimization process we randomly initialized the hyperparameter λ as follows: $\log\mu_0 \sim \text{Normal}(5, 2), \log\mu_1 \sim \text{Normal}(3, 2), \log\sigma_0 \sim \text{Normal}(2, 0.5), \log\sigma_1 \sim \text{Normal}(1.5, 0.5), \log\omega_k \sim \text{Normal}(3, 0.5)$, $\log\alpha \sim \text{Normal}(5, 0.5)$, and $\log\beta \sim \text{Normal}(2, 0.5)$. In total the learning algorithm need 54:34 minutes on a GPU machine (NVIDIA Tesla K40) to finish. The convergence diagnostics inspection shows successful convergence. Figure B.4.1 depicts a summary of convergence diagnostics.

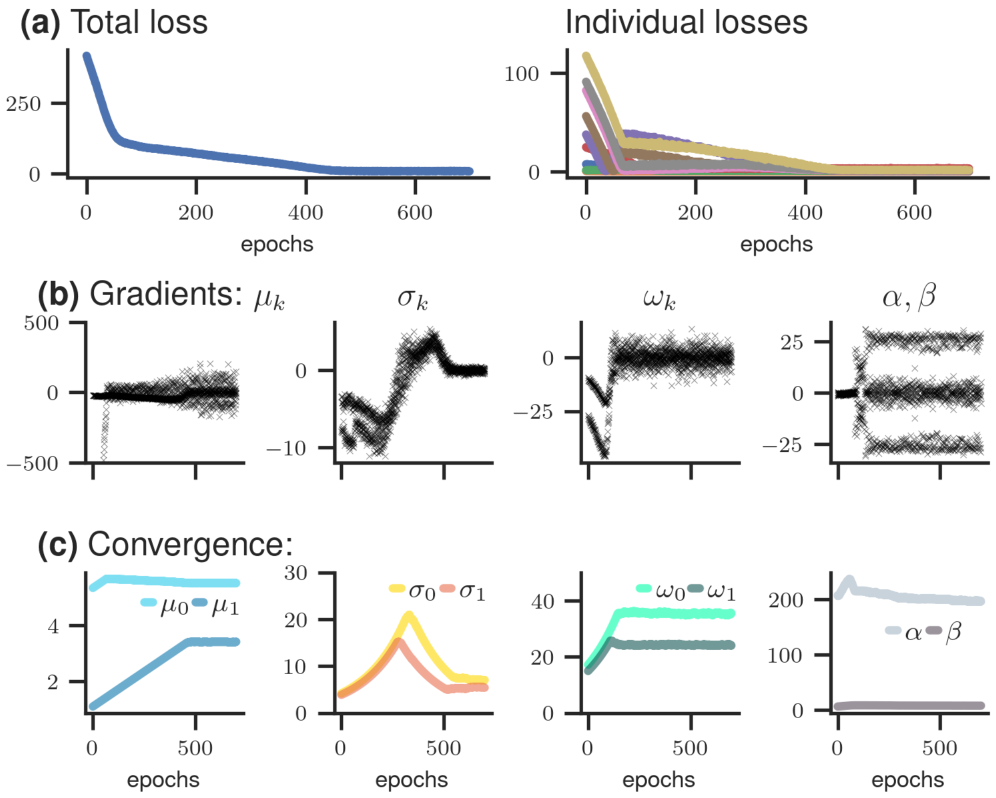

Figure B.4.1: *Convergence diagnostics for multilevel model with normal likelihood.* **(a)** loss value across epochs, demonstrating the desired decreasing trend of all loss values (i.e., total loss on the left as well as individual loss components on the right); **(b)** gradients of each learned hyperparameter λ; **(c)** learning of each hyperparameter across epochs.
